# Characterisation and preliminary functional analysis of N-acetyltransferase 13 from *Schistosoma japonicum*

**DOI:** 10.1186/s12917-021-03045-y

**Published:** 2021-10-22

**Authors:** Yalan Tang, Kerou Zhou, Qingqing Guo, Cheng Chen, Jing Jia, Qinghong Guo, Ke Lu, Hao Li, Zhiqiang Fu, Jinming Liu, Jiaojiao Lin, Xingang Yu, Yang Hong

**Affiliations:** 1 National Reference Laboratory for Animal Schistosomiasis, Shanghai Veterinary Research Institute, Chinese Academy of Agricultural Sciences, No.518 Ziyue Road, Minhang District, Shanghai, 200241 People’s Republic of China; 2 Key Laboratory of Animal Parasitology of Ministry of Agriculture, Shanghai Veterinary Research Institute, Chinese Academy of Agricultural Sciences, Shanghai, 200241 People’s Republic of China; 3 College of Life Science and Engineering, Foshan University, Foshan, 528231 People’s Republic of China

**Keywords:** *Schistosoma japonicum*, SjNAT13, N-acetyltransferase, RNAi, Development, Reproduction, Schistosome

## Abstract

**Background:**

N-acetyltransferase 13 (NAT13) is a probable catalytic component of the ARD1A-NARG1 complex possessing alpha (N-terminal) acetyltransferase activity.

**Results:**

In this study, a full-length complementary DNA (cDNA) encoding *Schistosoma japonicum* NAT13 (SjNAT13) was isolated from schistosome cDNAs. The 621 bp open reading frame of SjNAT13 encodes a polypeptide of 206 amino acids. Real-time PCR analysis revealed SjNAT13 expression in all tested developmental stages. Transcript levels were highest in cercariae and 21-day-old worms, and higher in male adult worms than female adult worms. The rSjNAT13 protein induced high levels of anti-rSjNAT13 IgG antibodies. In two independent immunoprotection trials, rSjNAT13 induced 24.23% and 24.47% reductions in the numbers of eggs in liver. RNA interference (RNAi) results showed that small interfering RNA (siRNA) Sj-514 significantly reduced SjNAT13 transcript levels in worms and decreased egg production in vitro.

**Conclusions:**

Thus, rSjNAT13 might play an important role in the development and reproduction of schistosomes.

**Supplementary Information:**

The online version contains supplementary material available at 10.1186/s12917-021-03045-y.

## Introduction

Schistosomiasis is a chronic and damaging zoonotic parasitic disease caused by trematode worms of the genus *Schistosoma*. It affects more than 200 million people in 52 countries across Africa, Asia and South America [1]. There are six types of mammalian schistosomes, and the most pathogenic towards human are *Schistosoma japonicum*, *S. mansoni* and *S. haematobium* [2, 3]. In China, the most common schistosome is *S. japonicum*.

Lysine acetylation, first discovered for histones, has been extensively studied in prokaryotes and eukaryotes [4–9]. It plays a critical role in the regulation of gene target gene expression [10, 11]. Protein amino-terminal acetylation influences cellular metabolism, and this ubiquitous post-translational modification is essential for the development of multicellular organisms [12, 13]. About 50–90% of synthesised proteins undergo amino-terminal acetylation in yeast, *Drosophila*, human and *Arabidopsis* [14–16]. However, only a few acetylated proteins have studied in schistosomes.

In our previous study, N-acetyltransferase 13 (NAT13) was found to be acetylated [8] and phosphorylated in 10-day old *S. japonicum* (unpublished), which indicates that this gene might play an important role in schistosomes. α-N-terminal acetylation is catalysed by different N-terminal acetyltransferases (NATs), in which the amino groups of protein N-termini and specific lysine residues accept an acetyl group from acetyl coenzyme A [17–19]. It is one of the most common protein covalent modifications in eukaryotes, with ~ 68% of yeast proteins and 85% of human proteins modified [20]. To date, six subtypes of amino-terminal acetyltransferases have been identified in eukaryotic cells (NatA-NatF), and all comprise more than one catalytic subunit [21, 22].

NAT13 is a probable catalytic component of the ARD1A-NARG1 complex possessing alpha (N-terminal) acetyltransferase activity. NAT13, also called San or N-acetyltransferase 5 (NAT5p), combines with the NatA subunits Naa10p and Naa15p to form NatE [17, 18, 23]. Naa50p acetylates a specific set of N termini, which differs from acetylation catalysed by the NatA activity of Naa10p, and it is defined as NatE even though physically associated with NatA [23–26]. In humans and *Drosophila*, this enzyme is important for chromosome resolution and segregation, and proper sister chromatid cohesion [26, 27]. Depletion of San causes premature sister chromatid separation in Hela cells, and San and its acetyltransferase activity are required for stable sister chromatid cohesion in metazoans [26].

In the present study, the SjNAT13 gene of *S. japonicum* was cloned, expressed, and expression levels were analysed at different developmental stages and in different sexes. The potential efficacy of SjNAT13 as a vaccine candidate against schistosome challenge was evaluated by schistosome infection of BALB/c mice. The functional roles of SjNAT13 in fecundity were evaluated by RNA interference (RNAi) in vitro and in vivo. The results expand our understanding of this enzyme in *S. japonicum*.

## Materials and methods

### Animals and parasites

Pathogen-free male BALB/c mice (6–8 weeks old, male) were purchased from Shanghai Experimental Animal Centre, Chinese Academy of Sciences. Snails (*Oncomelania hupensis*) infected by *S. japonicum* were maintained in Shanghai Veterinary Research Institute. Eggs were collected from the livers of mice 42 days after infection as described previously [28]. Miracidia were harvested from eggs after hatching in water for 1–2 h at 25 °C. Cercariae were collected by exposing snails infected with *S. japonicum* to light in water. Mice were infected with around cercariae through shaved abdominal skin. For euthanasia, mice were deeply anesthetized with CO_2_ and followed by cervical dislocation. Schistosomes were perfused from the hepatic portal system and mesenteric veins of the mice [29], which were percutaneously infected with *S. japonicum* 7, 14, 17, 21, 28, 35 and 42 days post-infection. Male and female schistosomes at 17, 21, 28, 35 and 42 days old were collected and separated manually.

### Quantitative real-time PCR (qPCR)

Total RNA from different developmental stages of schistosomes, including eggs, miracidia, cercariae, and 7, 14, 17, 21, 28, 35 and 42-day-old schistosomes was extracted with TRIzol reagent (Invitrogen, CA, USA) according to the manufacturer’s instructions. Total RNA concentrations were quantified by a NanoDrop 2000 instrument (Thermo Fisher Scientific, MA, USA). According to the standard protocol, total RNA was treated with gDNA Eraser (TaKaRa, Beijing, China) to remove genomic DNA before synthesising cDNA by RNA reverse transcription using a PrimeScript RT Reagent Kit (TaKaRa), and cDNAs were stored at − 20 °C until being used as templates for qPCR assays. Primers for the target gene SjNAT13 (GenBank accession no. FN317573.1) were 5′-TCATGTTGGCAATGAAGGCG-3′ (forward) and 5′-CCGAGTCGGATTCTCGTGTT-3′ (reverse). *S. japonicum* NADH-ubiquinone reductase served as an internal standard for normalisation [30], and primers for this gene were 5′-CGAGGACCTAACAGCAGAGG − 3′ (forward) and 5′-TCCGAACGAACTTTGAATCC − 3′ (reverse). All qPCR experiments were performed in a 20 μl reaction mixture containing 2 μl of cDNA, 10 μl of 2 × SYBR Primer Ex TagII (TaKaRa), 6 μl of H_2_O, 1.6 μl primers (forward and reverse, 10 μM each) and 0.4 μl ROX reference dye. All experiments were performed technically in triplicate. The relative mRNA expression level of SjNAT13 was calculated using the 2^-ΔΔCt^ method [31].

### Expression and purification of recombinant SjNAT13 protein (rSjNAT13)

Primers were designed according to the nucleotide sequences of *S. japonicum* NAT13. The ORF of SjNAT13 was amplified by PCR with forward (5′- TAGGATCCATGATTGCTGTACGTACCGA-3′) and reverse (5′-TAGAATTCTCAGTCAGATTCCGAGTCGG-3′) primers containing *Bam*HI and *Eco*RI restriction sites, respectively. The cDNAs of 21-day-old worms were used as template to amplify the target gene by PCR according the following amplification conditions: 94 °C for 3 min, followed by 30 cycles at 94 °C for 30s, 55 °C for 30 s, and 72 °C for 60 s, and a final extension at 72 °C for 5 min. PCR products were purified using a DNA gel extraction kit (Axygen, CA, USA) according to the manufacturer’s instructions. Purified PCR products were subcloned into the pMD19-T vector (TaKaRa) and the resulting recombinant plasmid pMD19-T-SjNAT13 was transformed into competent *Escherichia coli* (DH5α) cells. Successful construction of the recombinant plasmid was confirmed by antibiotic selection, and further corroborated by DNA sequencing.

The SjNAT13 gene fragment was digested from pMD19-T-SjNAT13 with *Bam*HI and *Eco*RI and subcloned into the prokaryotic expression vector pET28a(+). The resulting recombinant plasmid pET28a(+)-SjNAT13 was transformed into *E. coli* BL21 (DE3) cells (Tiangen, Beijing, China) and confirmed by DNA sequencing. Positive cells were grown in 500 ml of Luria-Bertani medium at 37 °C until the OD_600_ reached 0.6, then induced by adding isopropyl-1-thio-b-D-galactoside (IPTG) to a final concentration of 1 mM and culturing was continued at 37 °C for a further 6 h. Expression of the rSjNAT13 protein with a His-tag were boiled with loading buffer for 5 min using water bath and analyzed by sodium dodecyl sulfate polyacrylamide gel electrophoresis (SDS-PAGE). Gels were ran for 10 min under 80 V for 4% stacking gel and then for almost 60 min under 120 V for 12% resolving gel until the Bromophenol blue at the botom of the gel. Then, they were stained with 0.1% coomassie brilliant blue R-250 (25% (V/V) isopropyl alcohol, 10% (V/V) glacial acetic acid and water). The protein was purified by IDA-Nickel magnetic beads (Beaver, Jiangsu, China) according to the instructions.

### Evaluation of the immunoprotective efficacy of rSjNAT13

Thirty BALB/c mice were randomly divided into A, B and C groups with 9 mice per group. Group A was immunised three times subcutaneously with 20 μg rSjNAT13 emulsified with Montanide™ ISA206 (Seppic) at 2-week intervals. The emulsification condition was followed the instruction manual and references [32]. Group B (adjuvant control) and group C (blank control) were injected with phosphate-buffered saline (PBS) emulsified with adjuvant and PBS alone, respectively. At 2 weeks after the final immunisation, all mice were challenged percutaneously with 40 ± 1 viable cercariae. All mice were sacrificed and worms were perfused and counted at 42 days post-infection (dpi). The numbers of eggs was counted and the average eggs per gram of liver (EPG) was calculated. Finally, the EPG value resulting from each pair of schistosomes (EPG/pair) was calculated.

Immunoprotective efficacy was evaluated by calculating the rate of reduction in worm and egg counts as follows:

$${\displaystyle \begin{array}{l}\mathrm{Percentage}\ \mathrm{reduction}\ \mathrm{of}\ \mathrm{worm}\ \mathrm{burden}=\left(\mathrm{mean}\ \mathrm{worm}\ \mathrm{burden}\ \mathrm{of}\ \mathrm{control}\ \mathrm{group}-\mathrm{mean}\ \mathrm{worm}\ \mathrm{burden}\ \mathrm{of}\ \mathrm{vaccinated}\ \mathrm{group}\right)/\mathrm{mean}\ \mathrm{worm}\ \mathrm{burden}\ \mathrm{of}\ \mathrm{control}\ \mathrm{group}\times 100\%;\\ {}\mathrm{percentage}\ \mathrm{reduction}\ \mathrm{in}\ \mathrm{EPG}\ \mathrm{for}\ \mathrm{each}\ \mathrm{pair}\ \mathrm{of}\ \mathrm{schistosomes}=\left(\mathrm{mean}\ \mathrm{EPG}/\mathrm{pair}\ \mathrm{from}\ \mathrm{control}\ \mathrm{group}-\mathrm{mean}\ \mathrm{EPG}/\mathrm{pair}\ \mathrm{from}\ \mathrm{vaccinated}\ \mathrm{group}\right)/\mathrm{mean}\ \mathrm{EPG}/\mathrm{pair}\ \mathrm{from}\ \mathrm{control}\ \mathrm{group}\times 100\%.\end{array}}$$

All animal experiments were repeated in two independent trials.

### Detection of specific antibodies against rSjNAT13

Sera from each mouse were collected from the retro-orbital plexus before the first vaccination, 1 week after each vaccination, and before sacrifice. Sera were used to detect specific IgG antibodies against rSjNAT13 by ELISA. Briefly, a 96-well microtiter plate (Corning-Costar, MA, USA) was coated with 100 μl of rSjNAT13 (10 μg/ml) diluted in carbonate-bicarbonate buffer (pH 9.6) per well at 4 °C overnight. The plate was then washed three times with PBST (0.05% Tween 20 in PBS) and blocked with 200 μl of 0.5% gelatin per well in PBS at 37 °C for 1 h. After washing three times and diluting serum samples 1:100 in PBS, 100 μl of each serum sample was added and incubated for 2 h at 37 °C. After washing, 100 μl of HRP-conjugated goat anti-mouse IgG (1:4000 dilution; Beyotime, Shanghai, China) in PBS was then added and incubated at 37 °C for 1 h. PBST washes were performed three times after each step, with 5 min between each step. Plates were washed three times, and reactions were performed by adding the substrate 3,3′,5,5′-tetramethyl benzidine dihydrochloride (TMB; Tiangen) and incubating at room temperature in the dark for 5 min, then stopping with 2 M H_2_SO_4_ (50 μl/well). Finally, the absorbance was measured at 450 nm using a microplate reader (BioTek, VT, USA).

### Immunofluorescence localisation of SjNAT13 in *S. japonicum*

Fresh 28-day-old worms were collected and fixed in 4% formaldehyde solution at RT for 30 min. Worms were washed three times and permeabilised with proteinase K for 20 min at RT, and 5% bovine serum albumin was added and incubated for 30 min at RT as a blocking agent. After washing with PBST, samples were incubated at 4 °C overnight with anti-rSjNAT13 sera or native mice sera diluted 1:100. Samples were then incubated for 30 min with Alexa Fluor 555-labeled Donkey Anti-Mouse IgG (H + L) at a 1:500 dilution (Beyotime) in the dark. Finally, samples were stained with 2-(4-amidinophenyl)-6-indolecarbamidine dihydrochloride (DAPI) for 5 min, and sealed with Antifade Mounting Medium (Solarbio, Beijing, China). PBST was used to wash samples three times for 10 min each time, and slides were imaged using a Zeiss laser confocal microscope (Jena, Germany).

### Analysis of siRNA silencing of SjNAT13

The 42-day-old paired worms were collected and cultured in a 6-well flat bottom plate (15 pairs/well) in 3 ml culture medium at 37 °C in a humidified 5% CO_2_ incubator. Four siRNAs targeting SjNAT13 were designed based on the SjNAT13 gene sequence (Table 1), and each siRNA targeted a different coding sequence region of the gene. These siRNAs were designed and commercially synthesized by Shanghai GenePharma Co., Ltd. (Shanghai, China). To choose the most effective siRNA, five paired 42-day-old worms were placed in cuvettes containing 70 μL DMEM medium (Thermo Fisher Scientific) with 30 μl SjNAT13 siRNA (final concentration 12 μM) for electroporation [33, 34]. Control groups were treated with negative control siRNA (no homologous sequences in the schistosomal genome, NC group) or DEPC water (no siRNA, Blank group). After electroporation, the five paired worms were placed in a well and cultured in 3 ml culture medium at 37 °C for 72 h. Total RNA from each group of schistosomes was extracted and reverse-transcribed. SjNAT13 transcript levels were measured by qPCR and the effects of the four siRNAs on SjNAT13 were evaluated. All RNAi experiments were performed in triplicate independently.

**Table 1:** Sequences of SjNAT13-specific siRNAs and the control siRNA

Name	Sense	Antisense
siRNA-514	5′-GCGCAGUUGCUUUCUACAATT-3′	5′-UUGUAGAAAGCAACUGCGCTT-3′
siRNA-48	5′-GCUGUACGUACCGAACAAATT-3′	5′-UUUGUUCGGUACGUACAGCTT-3′
siRNA-154	5′-UCAAGCAGUUUAGACUGAUTT-3′	5′-AUCAGUCUAAACUGCUUGATT-3′
siRNA-575	5′-CCGACGCAUUCACCCUCAATT-3′	5′-UUGAGGGUGAAUGCGUCGGTT-3′
NC siRNA	5′-UUCUCCGAACGUGUCACGUTT-3′	5′-ACGUGACACGUUCGGAGAATT-3′

### Effects of RNAi in vitro and in vivo

Five 42-day-old paired worms were placed in cuvettes containing 70 μl DMEM medium (Gibco) with 30 μl siRNA-514 (final concentration 12 μM) for electroporation. Control groups were treated with negative control siRNA and DEPC water. After electroporation, schistosomes were transferred back to 6-well culture plates and incubated in a humidified 5% CO_2_ chamber at 37 °C for 72 h. Culture medium was changed every day and eggs were collected from each group and counted under light microscopy.

In this experiment, 15 mice were challenged with 40 ± 1 viable cercariae through shaved abdominal skin using the slide-cover-glass method and divided into three groups (five mice/group). At day 18 dpi, each mouse received four injections of 1 OD siRNA-514, NC siRNA, or 100 μl PBS via the tail vein every 6 days. At 42 dpi, worms were recovered from the hepatic veins by perfusion and counted. Livers were collected, the numbers of eggs was counted, and the EPG value in liver was calculated. Finally, the EPG value resulting from each pair of schistosomes (EPG/pair) were calculated.

### Statistical analysis

Data are expressed as mean ± standard deviation (SD) for at least three replicates of independent experiments performed under identical conditions. Statistical analysis was performed by analysis of variance (ANOVA) and independent-sample t-tests using GraphPad Prism 7.0, and *p* < 0.05 was considered statistically significant.

## Results

### Cloning, expression and purification of rSjNAT13

**Fig. 1:**
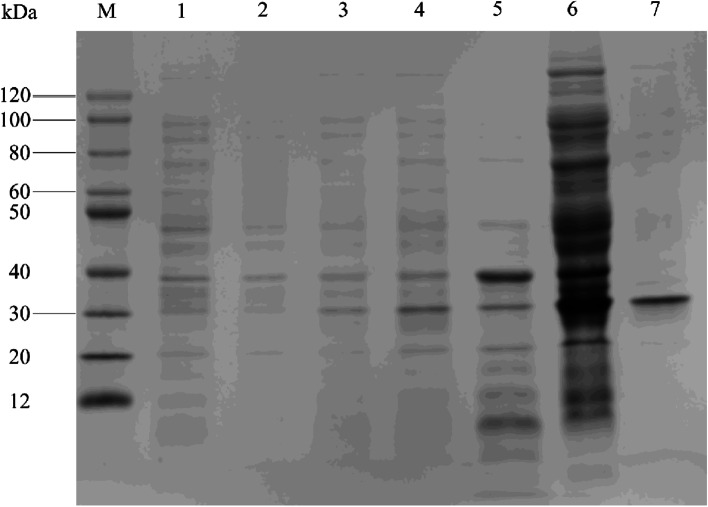
SDS-PAGE analysis of rSjNAT13. M, Protein markers; 1 and 2, total extract from a clone harbouring pET28a(+) before and after induction with 1 mM IPTG at 37 °C; 3 and 4, total extract from a clone harbouring pET28a(+)-SjNAT13 before and after induction with 1 mM IPTG at 37 °C; 5 and 6, supernatant and inclusion bodies of pET28a(+)-SjNAT13 after lysis; 7, purified rSjNAT13

The full-length sequence of SjNAT13 was obtained by RT-PCR amplification from cDNA of schistosomes. The 621 bp ORF encodes a protein of 206 amino acids. The rSjNAT13 protein containing a His-tag was successfully expressed in *E. coli* BL21 (DE3) cells induced by IPTG at 37 °C. The rSjNAT13 protein was expressed in inclusion bodies, and mostly solubilised in 8 M urea, but purification under denaturing conditions using magnetic His-Bind beads was unsuccessful. Purified rSjNAT13 was subjected to refolding by dialysis in PBS, and the molecular weight was ~ 30 kDa according to SDS-PAGE (Fig. 1).

### SjNAT13 mRNA profiles analysed by qPCR

**Fig. 2:**
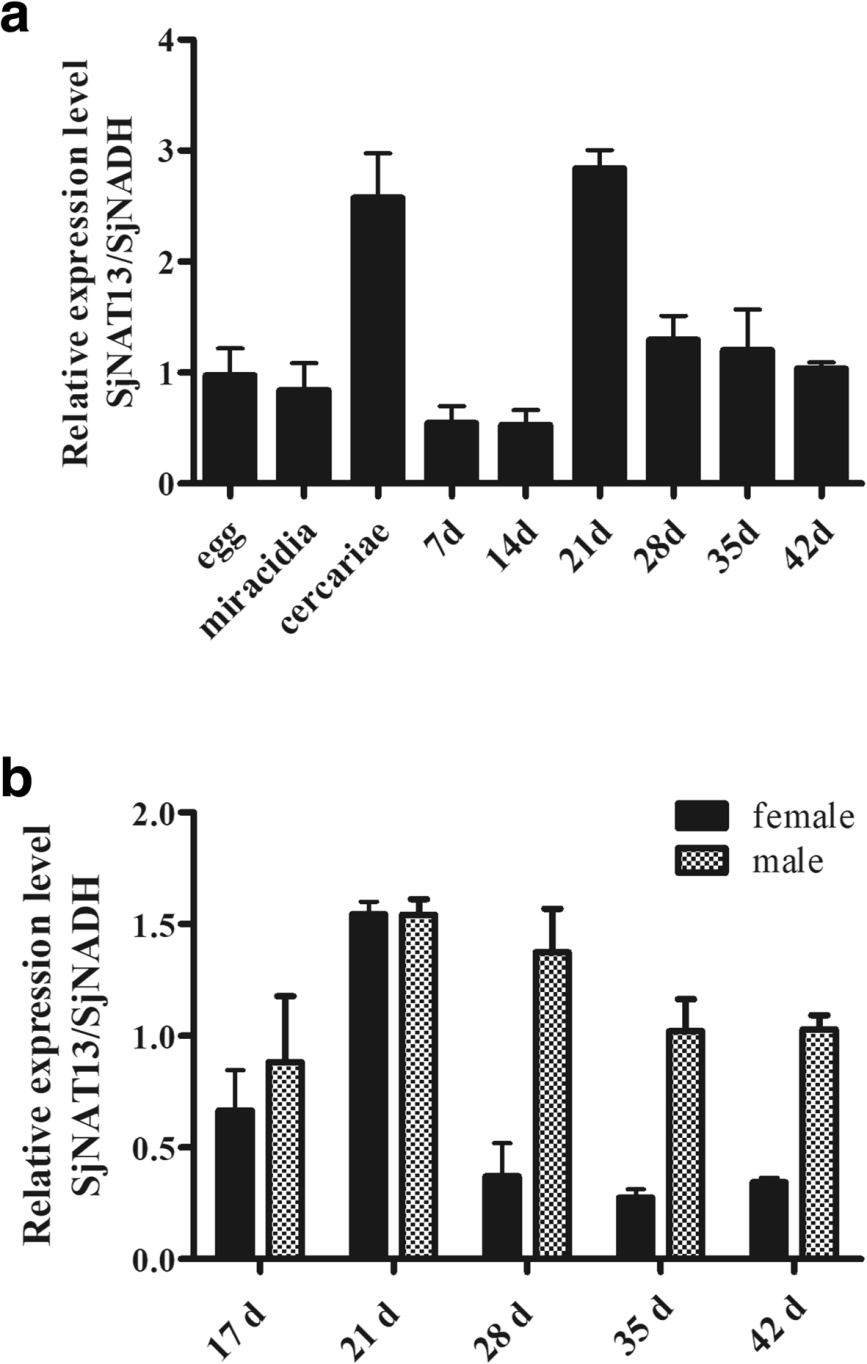
SjNAT13 mRNA profiles at different developmental stages, and in female and male worms. **a** Analysis of SjNAT13 expression levels at different stages of *S. japonicum*. **b** Analysis of SjNAT13 expression levels in different sexes at 17, 21, 24, 28, 35 and 42 days. Data are expressed as mean ± S.D. (*n* = 3)

Expression levels of SjNAT13 were measured in eggs, miracidia, cercariae, and in male and female 7-, 14-, 21-, 28-, 35- and 42-day-old schistosomes by qPCR. The results showed that the SjNAT13 gene was expressed in all developmental stages of schistosomes, and levels were higher in cercariae and 21-day-old worms than other stages, and significantly lower in 7- and 14-day-old schistosomula (Fig. 2a). In addition, expression levels of SjNAT13 in 28-, 35- and 42-day-old male worms were significantly higher than in female worms (Fig. 2b).

### Protective immune efficacy induced by rSjNAT13

The protective efficacy of rSjNAT13 in mice was evaluated, and the percentage reduction in worm burden and liver EPG were calculated (Table 2). Mice immunised with rSjNAT13 respectively displayed a 12.30 and 16.36% decrease in the number of worms compared with PBS controls. However, in the first trial, the adjuvant group experienced a higher worm reduction than the rSjNAT13 group compared with PBS controls. Compared with the worm reduction, the decrease in EPG in liver for each pair of worms was 24.23 and 24.47%, respectively, compared with PBS controls in two independent trials.

**Table 2:** The results of protective efficacy against *S. japonicum* challenge in BALB/c mice induced by immunized with rSjNAT13

Group	Worm burden (mean ± S.D.)	Percent reduction in worm burden (%)	EPG (mean ± S.D.)	Percent reduction in liver egg count (%)
Experiment 1				
A	11.89 ± 4.40^a^	12.30%	38,278.03 ± 7290.86^a^	24.23%
B	11.22 ± 5.85^a^	17.21%	42,762.48 ± 24,502.99^a^	15.35%
C	13.56 ± 3.74^a^	–	50,519.28 ± 17,421.34^a^	–
Experiment 2				
A	10.22 ± 2.11^a^	16.36%	45,603.10 ± 9503.84^a^	24.47%
B	11.89 ± 2.52^a^	2.73%	54,459.61 ± 13,764.93^ab^	9.81%
C	12.22 ± 2.33^a^	–	60,377.89 ± 16,524.13^b^	–

Data are expressed as mean ± S.D. Values with different superscripts in the same column differ significantly (*P* < 0.05). Values with same superscripts in the same column do not differ significantly (*P* > 0.05)

### Detection of rSjNAT13-specific IgG antibodies and immunolocalisation analyses

**Fig. 3:**
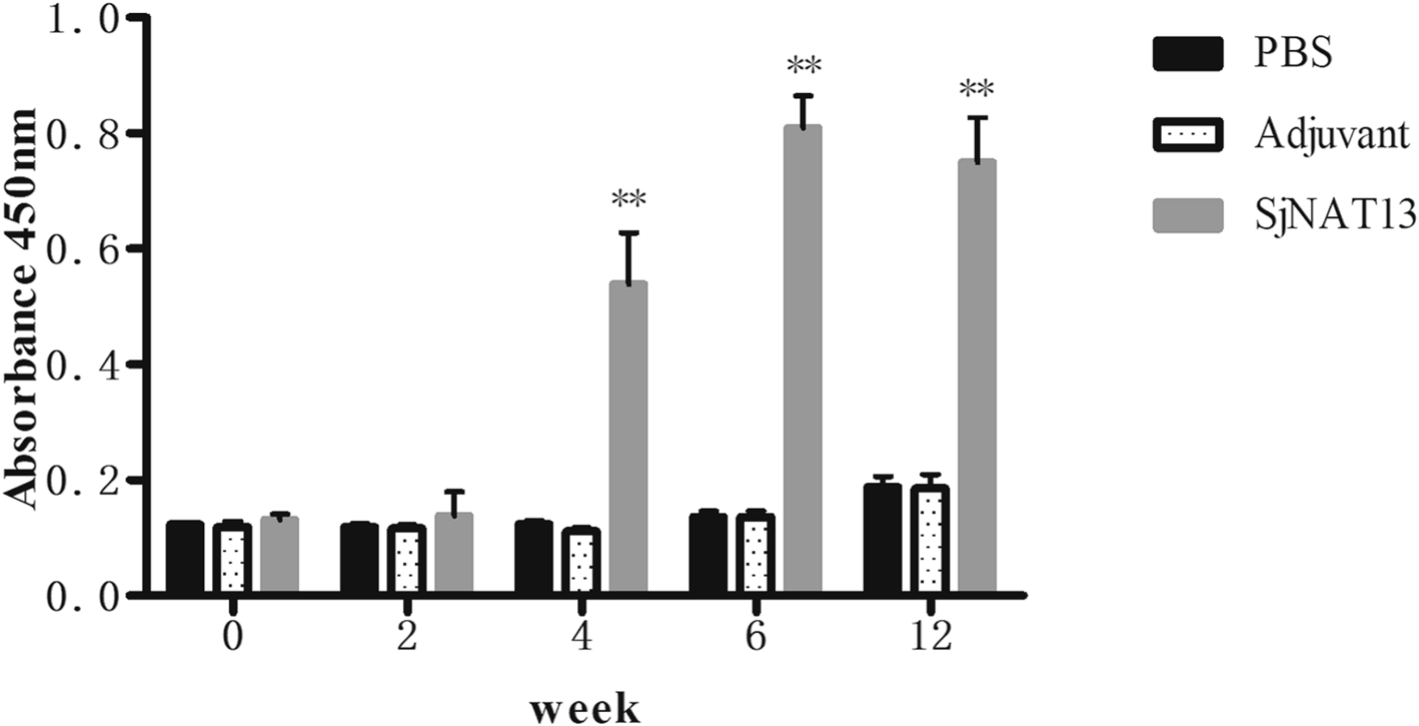
Specific IgG antibody response to rSjNAT13 in different treatment groups assessed by ELISA. Mice were injected subcutaneously with rSjNAT13, 206 adjuvant, or PBS, and sera were collected and analysed by ELISA. The asterisks (**) indicate significantly increased serum antibody titers compared with the PBS group (*P* < 0.01)

**Fig. 4:**
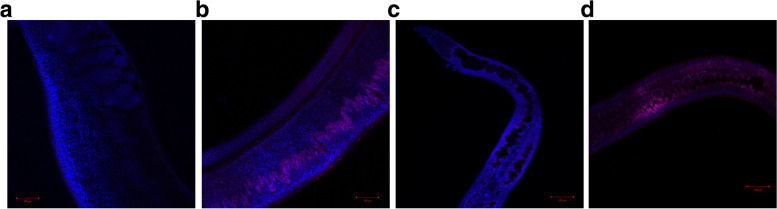
Localisation analysis of SjNAT13 by immunofluorescence*.* The sections of male (**A**) and female (**C**) worms were probed with normal mice sera. The sections of male (**B**) and female (**D**) worms were probed with SjNAT13-specific mice sera. Secondary antibody (Alexa Fluor 555-labeled donkey anti-mouse IgG, red) was used for immunofluorescence detection of SjNAT13 on schistosomes. DAPI (blue) was used to stain parasite nuclei

Serum samples from each immunoprotection test group were evaluated to measure the levels of IgG antibodies specific to rSjNAT13 by ELISA (Fig. 3). Compared with the 206 adjuvant and PBS control groups, the rSjNAT13 vaccination group displayed a dramatic increase in specific anti-rSjNAT13 IgG antibodies after the second vaccination, and this was further increased with administration of a third injection, and maintained at a high level until animals were sacrificed. There were no significant differences in specific IgG levels between 206 adjuvant and PBS groups, and levels maintained low in both. The distribution of SjNAT13 in 28-day-old schistosomes was investigated by immunofluorescence assay using the prepared anti-rSjNAT13 sera. The results showed that SjNAT13 was distributed widely and systemically in schistosomes, and mainly expressed in the tegument and parenchyma under the body wall musculature (Fig. 4).

### Efficacy of RNAi silencing of SjNAT13 in vitro and in vivo

**Fig. 5:**
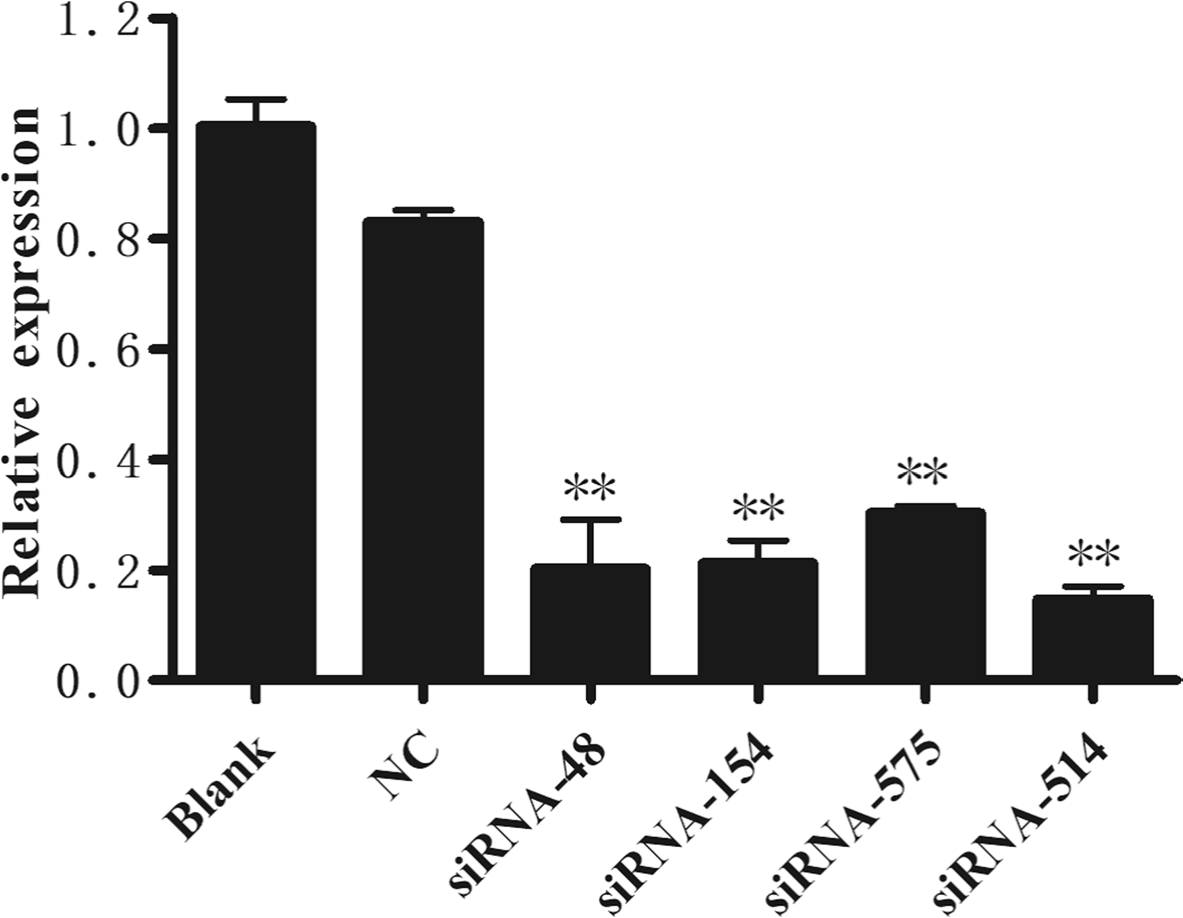
Effects of different siRNAs on SjNAT13 in vitro. Comparison of the effects on SjNAT13 gene expression in negative control (NC) and four siRNA groups (siRNA-48, siRNA-154, siRNA-575 and siRNA-514). The asterisks (**) indicate significantly decreased of SjNAT13 expression compared with the NC group (*P* < 0.01, *n* = 3)

The RNAi results indicated that SjNAT13 mRNA levels were reduced by all four siRNAs; SjNAT13 transcripts in worms treated with siRNA-514, siRNA-48, siRNA-154 and siRNA-575 were respectively reduced by 87.74, 73.48, 74.48 and 70.13% compared to the blank group, and 69.63, 55.36, 56.37 and 52.02% compared to the NC group (Fig. 5). Thus, siRNA-514 was chosen as the optimal siRNA for further RNAi experiments. After electroporation with siRNA-514 in vitro, there was no obvious difference in the vitality of schistosomes by the naked eye. However, the spawning ability of the siRNA-514 group was decreased ~ 38.91% (*p* < 0.05) compared with the blank group (Table 3).

**Table 3:** The effect of SjNAT13 interference on the fertility of *S. japonicum* in vitro

Group	Egg number (mean ± S.D.)	Egg reduction (%)
Blank	16,283.33 ± 2332.37^a^	–
NC	14,737.50 ± 2088.04^a^	9.49%
siRNA-514	9947.92 ± 1113.94^b^	38.91%

Values with different superscripts in the same column differ significantly (*P* < 0.05). Values with same superscripts in the same column do not differ significantly (*P* > 0.05)

In the in vivo RNAi experiment, there were no obvious changes in the vitality or development of schistosomes in the three groups. Compared with the blank group, NC and siRNA-514 groups showed a 16.36% (*p* > 0.05) and 10.91% (*p* > 0.05) decrease in the number of worms, respectively. However, the in vivo RNAi experiment revealed a decrease in the reproductive capacity and survival of paired adult schistosomes. The EPG values in liver for each pair of worms in NC and siRNA-514 groups were decreased by 0.80% (*p* > 0.05) and 37.66% (*p* < 0.05), respectively, compared with the blank group (Table 4). Thus, RNAi silencing of SjNAT13 in vivo could influence the reproductive capacity of paired schistosomes and lower the egg burden in the mouse liver.

**Table 4:** The results of SjNAT13 interference on *S. japonicum* in vivo

Group	Worm burden (mean ± S.D.)	Percent reduction in worm burden (%)	EPG/pair (mean ± S.D.)	Percent reduction in liver egg count (%)
Blank	11.0 ± 3.32^a^	–	12,768.45 ± 2727.10^a^	–
NC	9.2 ± 2.28^a^	16.36%	12,666.13 ± 1609.43^a^	0.80%
siRNA-514	9.8 ± 3.03^a^	10.91%	7959.99 ± 1172.58^b^	37.66%

Values with different superscripts in the same column differ significantly (*P* < 0.05). Values with same superscripts in the same column do not differ significantly (*P* > 0.05)

## Discussion

N-terminal acetylation is one of the most common covalent modifications of proteins. It takes place on ribosomes and is catalysed by a family of N-terminal acetyltransferases (NATs) [25]. Naa50 is the catalytic subunit of NatE, a highly conserved NAT with a classical GNAT fold that transfers an acetylate group to the α-amino group of the N terminus of proteins that retain the initiating methionine, thereby regulating genome integrity [35, 36]. In metazoans, Naa50 is essential for sister chromatid cohesion and chromosome condensation, and blocking the enzyme disrupts sister chromatid cohesion and causes premature separation of sister chromatids and mitotic arrest [26, 36, 37]. Proper chromosome segregation is indispensable for the correct transmission of genetic information [36]. If this process fails aneuploidy can occur, potentially leading to tumorigenesis and other human diseases [38–40]. Naa50 is believed to regulate the correct interaction between the cohesion subunits Scc1 and Smc3 during *Drosophila* wing development [13]. Recent research discovered that human Naa50 negatively regulates microtubule polymerisation via the internal acetylation of beta-tubulin [41].

The results of qPCR analysis showed that SjNAT13 was highly expressed in the cercariae stage. In this stage, schistosome cercariae prepare to infect their specific host and in doing so they penetrate the skin, shed their tails, and transform into schistosomula. This process involves shedding of the cercarial surface membranes, replacement with preformed material from subtegumental cell bodies, and altering glucose catabolic pathways [42, 43]. Thus, schistosomes synthesise numerous proteins to achieve these major morphological changes and adapt to the new living environment with altered temperature, sugar concentration and osmolarity. Many of these proteins function in different pathways and perform a variety of functions following post-translational modification such as acetylation and phosphorylation. This might be one of the reasons for up-regulation of SjNAT13 in cercariae.

After cercariae invade the final host by penetrating the skin, they transform into schistosomula, migrate within the bloodstream and undergo further developmental changes. Our qPCR results also revealed that expression of SjNAT13 in schistosomes was lower at 7 and 14 days, but it was increased at 17 days when worms began to pair. Moreover, this gene was expressed at comparable levels in male and female worms at 17 and 21 days. However, expression levels of SjNAT13 in male worms were 3–4 times higher than in female worms at 28, 35 and 42 days. In *S. japonicum*, male and female worms pair at 16–17 dpi, then become sexually mature. Worms experience a period of rapid growth and significant changes in morphology, during which many new cells and proteins are produced. Thus, NATs are needed for normal cell division and protein stability. In previous studies, Naa50/San was found not to be necessary for mitosis in female germ-line stem cells of *Drosophila*, which might be a reason why it was less abundant in adult females than in adult male, but the exact reason remains unclear [27]. Lysine acetylation is thought to be essential for sperm motility and fertilisation in human [44]. Meanwhile, NAT2 participates in the detoxification of toxic arylamines, aromatic amines and hydrazines, and it is a novel genetic marker for susceptibility to idiopathic male infertility [45]. Thus, this gene might be associated with sexually mature schistosomes, especially for male worms.

In two independent immunoprotection experiments, mice immunised three times with rSjNAT13 produced higher levels of specific anti-rSjNAT13 IgG antibodies. However, the immunoprotection effects on worm burden were less than 20% different from PBS controls. Even so, the EPG value in liver caused by each pair of worms was steadily reduced by almost 25% in the two experiments. In trial 1, the adjuvant only group also caused a higher reduction in worm burden, but there was no significant difference among these three groups (*P* > 0.05). This might also be non-specific lung inflammation likely due to the short interval between final immunisation and cercarial challenge [46].

In order to further analyse the influence of this gene on schistosomes, in vitro and in vivo RNAi experiments were employed. Although there was no obvious difference in the vitality of schistosomes in RNAi experiments in vitro, the reduction in eggs for each pair of schistosomes was significantly decreased in the siRNA-514 group. A similar phenomenon was also apparent for in vivo RNAi experiments. Based on these results, SjNAT13 might be unsuitable as a vaccine candidate for directly reducing the worm burden in the final host. However, it had a strong effect on egg reduction, which could reduce host pathological injury, and also reduce disease transmission. We speculate that this effect might be caused by influencing sister chromatid cohesion and cell division.

An effective schistosome vaccine could be a useful tool for controling schistosomiasis [47]. An effective vaccine combined with praziqutal treatment might provide a good long-term strategy for controlling this disease [48]. In addition, eggs deposited in host tissues are the principal cause of pathology. Thus, a significant reduction in the fecundity of worms or the viability eggs could be very important for controlling schistosomiasis.

## Conclusions

In this study, SjNAT13 was cloned, expressed and characterised. The results showed that SjNAT13 displayed good immunogenicity and could induce the production of specific antibodies. Although the decrease in worm burden during schistosome infection was not significant, SjNAT13 caused a considerable reduction in the number of eggs in the liver, indicating potential value as a therapeutic target for reducing the reproduction of *S. japonicum* and controlling schistosomiasis.

## Supplementary Information

**Additional file 1.**

## Data Availability

Data supporting the conclusions of this article are included within the article.

## Abbreviations

ORF: Open reading frame
qPCR: Quantitative real-time PCR
cDNA: Complementary DNA
EPG: Eggs per gram
ELISA: Enzyme-linked immunosorbent assay
HRP: Horseradish peroxidase
RNAi: RNA interference
SDS: Sodium dodecyl sulfate
PBS: Phosphate-buffered saline
RT: Room temperature
