# Supplementary material for: Characterisation and preliminary functional analysis of N-acetyltransferase 13 from Schistosoma japonicum
Source: BMC Vet Res. 2021 Oct 22;17:335. doi: 10.1186/s12917-021-03045-y (PMC8540080; doi:10.1186/s12917-021-03045-y)
Supplement: Supplementary file 1 — Additional file 1. [file 12917_2021_3045_MOESM1_ESM.docx]

1 2 3 4 5 6 7 8 9


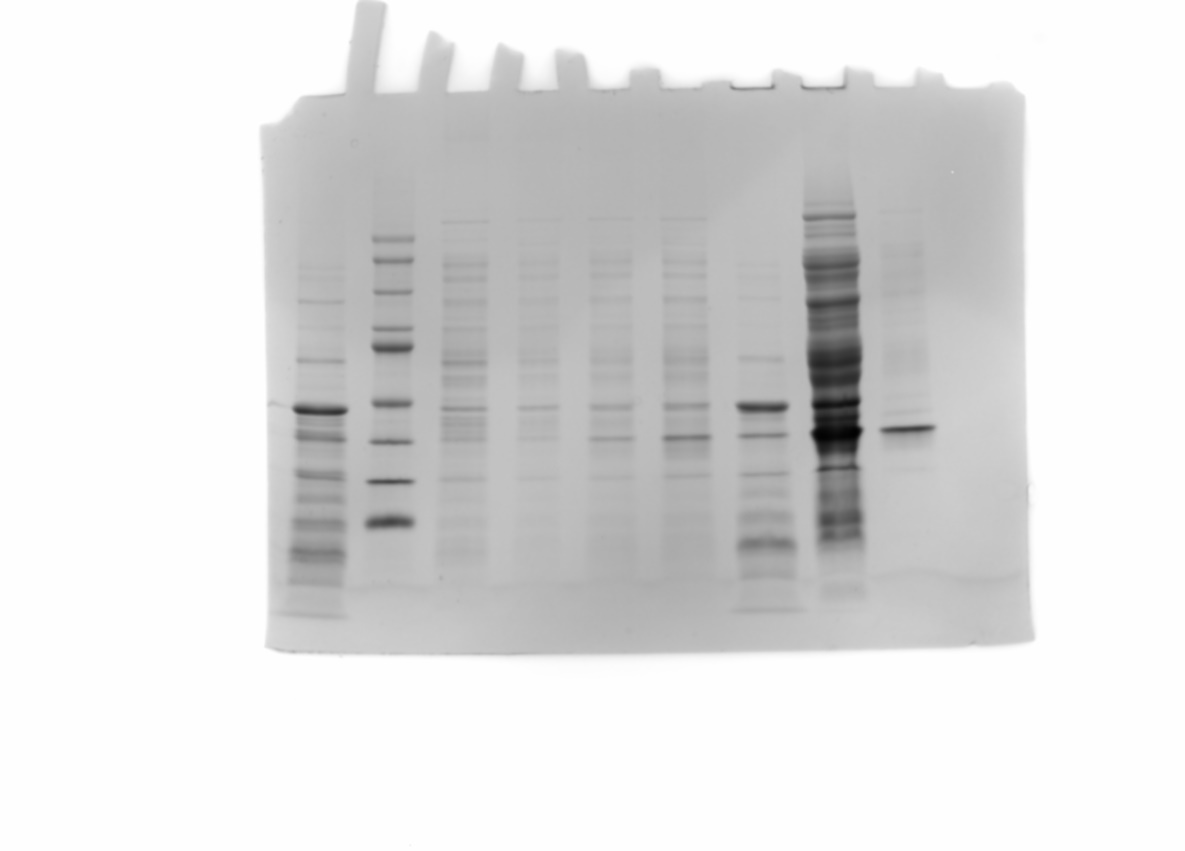


The the original, uncropped gel image of Fig. 1

2, Protein markers; 3 and 4, total extract from a clone harbouring pET28a(+) before and after induction with 1 mM IPTG at 37°C; 5 and 6 total extract from a clone harbouring pET28a(+)-SjNAT13 before and after induction with 1 mM IPTG at 37°C; 7 and 8 supernatant and inclusion bodies of pET28a(+)-SjNAT13 after lysis; 9, purified rSjNAT13.
